# *ATAD3A* variants manifest multisystematically in the brain, nerves, eyes, heart, liver, and skin

**DOI:** 10.1016/j.ymgmr.2020.100569

**Published:** 2020-01-25

**Authors:** Josef Finsterer, Rahim Aliyev

**Affiliations:** a Neurological Department, Krankenanstalt Rudolfstiftung, Messerli Institute, Vienna, Austria; b Department of Neurology and Clinical Neurophysiology, Azerbaijan State Advanced Training Institute for Doctors named after A. Aliyev, Baku, Azerbaijan

**Keywords:** Mitochondrial disorder, Pontocerebellar hypoplasia, ATAD3A, Multisystem, Cardiac involvement, Phenotype

In a recent article, Peralta et al. reported about 4 siblings from consanguineous parents with neonatal cerebellar hypoplasia, seizures, axial hypotonia, hypertrophic cardiomyopathy, hepatomegaly, congenital cataract, and facial dysmorphism due to the novel, homozygous missense variant c.1217T>G (p.Leu406Arg) in *ATAD3A* [1]. The study evokes the following concerns.

We do not agree with the notion that hypertrophic cardiomyopathy and hepatomegaly “expand the phenotypic spectrum commonly associated with pathogenic *ATAD3A* variants” [1]. At least hypertrophic cardiomyopathy has been reported previously in 2 of 8 unrelated individuals carrying the variant c.1582C>T in *ATAD3A* [2]. One of the 8 individuals also presented with right ventricular hypertrophy. Hypertrophic cardiomyopathy has been also reported in one of 6 patients carrying deletions of the *ATAD3A* and *ATAD3B* gene respectively [3]. The only phenotypic features which have not been reported previously are hepatomegaly and low plasma cholesterol [1],

The presence of hepatomegaly in all four siblings is not surprising given the fact that all four had cardiomyopathy. Assuming that all four had heart failure from cardiomyopathy, enlargement of the liver due to congestion is not unusual. We thus should know if the four siblings presented with other clinical features of heart failure (edema, neck vein distension), if systolic function was reduced, and if proBNP values were increased. In case hepatomegaly was not attributable to heart failure, we should know if there was steatosis or accumulation of other material on autopsy, or liver toxicity from drugs.

Features which have been previously reported but not in the present study include fetal distress [2,3], feeding difficulties [2], optic atrophy [2], peripheral spasticity [2,4], and axonal neuropathy [2,4]. We should know what is meant with “peripheral spasticity” and if these features were truly absent or if the authors not systematically looked for them,

This interesting study may profit from addressing the points mentioned above.

## Funding

No funding was received.

## Author contribution

JF: design, literature search, discussion, first draft, critical comments.

## Declaration of Competing Interest

There are no conflicts of interest.

## References

[1] Peralta S., González-Quintana A., Ybarra M., Delmiro A., Pérez-Pérez R., Docampo J., Arenas J., Blázquez A., Ugalde C., Martín M.A. (2019). Novel ATAD3A recessive mutation associated to fatal cerebellar hypoplasia with multiorgan involvement and mitochondrial structural abnormalities. Mol. Genet. Metab..

[2] Harel T., Yoon W.H., Garone C., Gu S., Coban-Akdemir Z., Eldomery M.K., Posey J.E., Jhangiani S.N., Rosenfeld J.A., Cho M.T., Fox S., Withers M., Brooks S.M., Chiang T., Duraine L., Erdin S., Yuan B., Shao Y., Moussallem E., Lamperti C., Donati M.A., Smith J.D., McLaughlin H.M., Eng C.M., Walkiewicz M., Xia F., Pippucci T., Magini P., Seri M., Zeviani M., Hirano M., Hunter J.V., Srour M., Zanigni S., Lewis R.A., Muzny D.M., Lotze T.E., Boerwinkle E., Baylor-Hopkins Center for Mendelian Genomics, University of Washington Center for Mendelian Genomics, Gibbs R.A., Hickey S.E., Graham B.H., Yang Y., Buhas D., Martin D.M., Potocki L., Graziano C., Bellen H.J., Lupski J.R. (2016). Recurrent De Novo and biallelic variation of ATAD3A, encoding a mitochondrial membrane protein, results in distinct neurological syndromes. Am. J. Hum. Genet..

[3] Desai R., Frazier A.E., Durigon R., Patel H., Jones A.W., Dalla Rosa I., Lake N.J., Compton A.G., Mountford H.S., Tucker E.J., Mitchell A.L.R., Jackson D., Sesay A., Di Re M., van den Heuvel L.P., Burke D., Francis D., Lunke S., McGillivray G., Mandelstam S., Mochel F., Keren B., Jardel C., Turner A.M., Ian Andrews P., Smeitink J., Spelbrink J.N., Heales S.J., Kohda M., Ohtake A., Murayama K., Okazaki Y., Lombès A., Holt I.J., Thorburn D.R., Spinazzola A. (2017). ATAD3 gene cluster deletions cause cerebellar dysfunction associated with altered mitochondrial DNA and cholesterol metabolism. Brain.

[4] Cooper H.M., Yang Y., Ylikallio E., Khairullin R., Woldegebriel R., Lin K.L., Euro L., Palin E., Wolf A., Trokovic R., Isohanni P., Kaakkola S., Auranen M., Lönnqvist T., Wanrooij S., Tyynismaa H. (2017). ATPase-deficient mitochondrial inner membrane protein ATAD3A disturbs mitochondrial dynamics in dominant hereditary spastic paraplegia. Hum. Mol. Genet..

